# Comparison of Four Extraction Techniques for the Evaluation of Volatile Compounds in Spray-Dried New Zealand Sheep Milk

**DOI:** 10.3390/molecules24101917

**Published:** 2019-05-18

**Authors:** Ryan High, Phil Bremer, Biniam Kebede, Graham T. Eyres

**Affiliations:** Department of Food Science, University of Otago, PO Box 56, Dunedin 9054, New Zealand; ryan.high@postgrad.otago.ac.nz (R.H.); phil.bremer@otago.ac.nz (P.B.); biniam.kebede@otago.ac.nz (B.K.)

**Keywords:** sheep milk, volatiles, flavour, lactone, extraction method

## Abstract

Recent growth and diversification of sheep milk products means more sophisticated methods are required to ensure their flavour quality. The objective of this study was to compare four extraction techniques for the analysis of volatile compounds in sheep milk by gas chromatography-mass spectrometry (GC-MS). Solvent Assisted Flavour Evaporation (SAFE), Solid Phase Microextraction (SPME), Headspace Sorptive Extraction (HSSE) and Stir Bar Sorptive Extraction (SBSE) were evaluated for their sensitivity, selectivity, reproducibility, and overall efficiency. A total of 48 volatile compounds from nine compound classes were identified in the spray-dried sheep milk. Alcohols, aldehydes, alkanes, carboxylic acids, ketones, lactones, sulphur compounds, nitrogen compounds, and terpenes were all present, but the differences between the methods were most apparent for lactones. SBSE extracted eight lactones, SAFE extracted four lactones and HSSE and SPME only detected trace levels of two lactones. Six of the lactones—δ-hexa-lactone, δ-octalactone, γ-decalactone, γ-dodecalactone, δ-tetradecalactone, and δ-hexadeca-lactone—were identified for the first time in spray-dried sheep milk. The present work demonstrated that SBSE is an effective tool for the extraction and analysis of volatiles, especially lactones, in sheep milk and dairy products in general. A discussion of the benefits and limitations of each method is included.

## 1. Introduction

Sheep milk, at around 1.5%, represents a relatively small proportion of global milk production, but its popularity has grown in recent decades [1]. From 1994 to 2013 global sheep milk production grew by 26%, led by dramatic increases in Africa and Asia [1]. As the demand for sheep milk increases and the dairy industry utilizes sheep milk for purposes beyond cheese and other traditional dairy products, it will also need methods to ensure the flavour quality of those products. As noted by Karagül-Yüceer, Cadwallader, and Drake, flavour is one of the most important attributes of a dairy product [2].

Excluding work on cheese, a limited amount of literature has directly examined the volatile flavour compounds and the impact of processing on those compounds in sheep milk. Teng et al. investigated the impact of seasonal variation, pasteurization, and spray-drying on the volatile branched chain fatty acids of New Zealand sheep milk [3]. The study found that there were seasonal effects and that spray-drying led to significantly higher levels of those fatty acids [3]. To the best of our knowledge, no studies have investigated the broader range of volatile compounds in spray-dried sheep milk or in New Zealand sheep milk. 

It is widely acknowledged that there is no perfect volatile extraction technique and when selecting a method, it is important to understand the specific advantages and shortcomings. Previous investigations of the volatile attributes of sheep milk have employed both headspace and more traditional distillation and liquid extraction techniques [4,5,6,7,8,9,10]. A benefit of Solid Phase Microextraction (SPME) has been the range of commercially available sorbent phases that can be tailored to selectively recover volatiles of interest. SPME was previously employed to examine the volatiles of sheep milk by Cais-Sokolinska et al. as well as Vasta at al. [8,10]. Cais-Sokolinska et al. identified 36 volatile compounds using a carboxen/polydimethylsiloxane (CAR/PDMS) fibre in sheep milk [8]. Similarly, Vasta et al. identified 37 volatile compounds across eight compound classes, including alcohols, aldehydes, ketones, furans, hydrocarbons, organic acids, terpenes, and sulphur compounds using a divinylbenzene/carboxen/polydimethylsiloxane (DVB/CAR/PDMS) fibre [10]. Addis et al. applied Dynamic Headspace (DHS) to milk from sheep that were fed different diets and identified 22 compounds that included ketones, aldehydes, alcohols, an ester, some hydrocarbons and terpenes [9]. In another series of studies, sheep milk volatiles were evaluated by low pressure distillation and liquid-liquid extraction and 67 volatile compounds in raw ovine milk were identified [4,5,6]. The volatiles identified by Moio et al. spanned nine compound classes and included esters, aldehydes, ketones, alcohols, sulphur compounds, lactones, nitrogen compounds, aromatic compounds and a few unknown compounds [4,5,6]. It was found by CHARM analysis that esters and aldehydes, along with dimethyl sulphone and indole were potent aroma compounds in raw milk [5]. Moio, Langlois, and Etievant postulated the importance of lactones as an aroma-active compound class in the milk but limited recovery of the lactones by their equipment prevented that conclusion to made at the time [5]. 

The volatile compounds in sheep milk are similar to those found in bovine and other species’ milks [5,11]. The literature addressing bovine milk volatiles is more extensive than for sheep milk and additional isolation techniques have been tested that could prove useful for the evaluation of sheep milk. In a direct comparison of bovine milk volatiles extracted by DHS and SPME, SPME was found to have better reproducibility and similar sensitivity [12]. Indeed, SPME has proved a popular and successful technique for the extraction of milk volatiles and enjoys widespread application to fluid and spray-dried milk samples [12,13,14,15,16,17,18,19,20]. Modern distillation techniques like Solvent Assisted Flavour Evaporation (SAFE) have also been employed extensively for the extraction of milk volatiles [2,11,20,21,22,23]. Another set of techniques, known as Headspace Sorptive Extraction (HSSE) and Stir Bar Sorptive Extraction (SBSE), utilise a polydimethylsiloxane (PDMS) extraction phase on a magnetic stir bar to extract volatile compounds from either the headspace or from a liquid sample. These methods have been applied to human breast milk for the identification of odour-active volatiles [24]. In contrast with SPME, SBSE and HSSE may be more sensitive due to the larger ratio between the sorbent phase and the sample, specifically 63 µL of PDMS on a 10 mm x 1 mm stir bar compared with ~0.5 µL on a SPME fibre [25]. However, the single phase PDMS stir bars may selectively discriminate against small polar compounds [25,26]. SBSE has demonstrated good overall sensitivity, especially for larger, less volatile analytes, such as lactones [24]. Given their successful applications in other milk types, the SAFE, HSSE, and SBSE techniques could prove useful for the evaluation of sheep milk volatiles. 

The objective of this study was to adapt four volatile extraction techniques to sheep milk and compare their respective benefits and biases. The experiment investigated the sensitivity, selectivity, and reproducibility of SAFE, SPME, SBSE, and HSSE techniques for the analysis of volatile compounds from spray-dried New Zealand sheep milk. Consideration was also given to the efficiency of each method, though the authors acknowledge the subjectivity of this attribute due to the spectrum of available equipment and laboratories. This paper will provide insight into the benefits and limitations of the four extraction techniques for the evaluation of sheep milk volatiles and will expand knowledge of the volatile compounds in spray-dried sheep milk beyond the branched chain free fatty acids. 

## 2. Results

### 2.1. Evaluation Criteria

**Figure A1:**
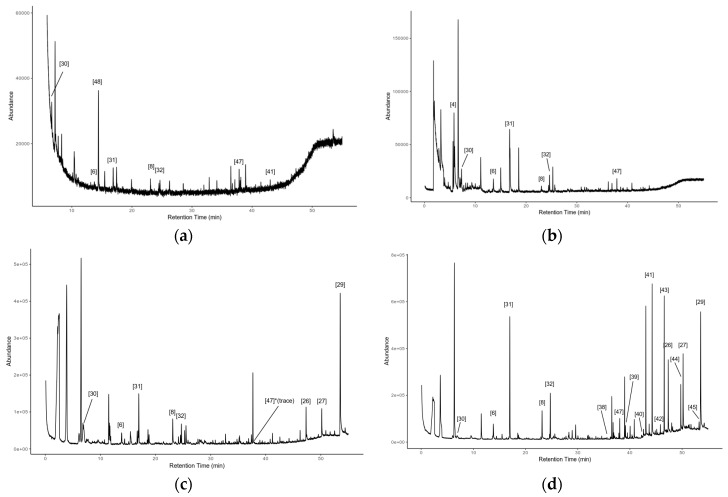
Representative Total Ion Chromatogram of (**a**) Solvent Assisted Flavour Evaporation (SAFE), (**b**) Solid Phase Microextraction (SPME), (**c**) Headspace Sorptive Extraction (HSSE), and (**d**) Stir Bar Sorptive Extraction (SBSE) extracts from sheep milk. Selected peaks labelled with numbers corresponding to Table 1.

To evaluate the extraction methods, the sensitivity, selectivity, reproducibility, and overall efficiency was compared. The sensitivity of the methods was evaluated by the overall apparent concentration of volatiles detected. The selectivity of the methods was assessed based on the apparent volatile concentration detected by compound class. The reproducibility was assessed on the overall average reproducibility of each method and within each compound class. For the purpose of this paper, efficiency has been defined as the time required to prepare the necessary equipment, glassware and samples for extraction and the time required to perform the extraction. Representative total ion chromatograms for each of the methods can be found in Figure A1 of Appendix A.

### 2.2. Sensitivity and Selectivity

**Figure 1:**
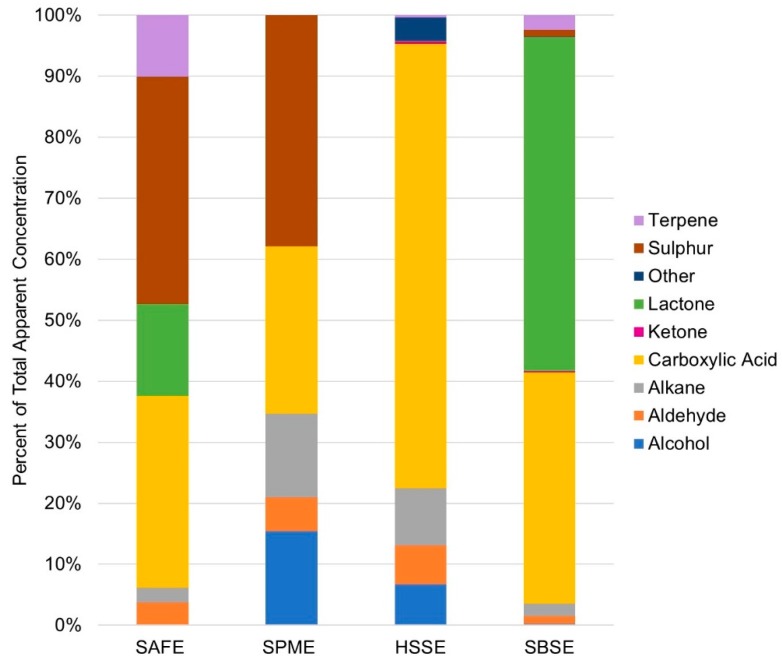
The percentage distribution of volatile compounds in each compound class detected in sheep milk by Solvent Assisted Flavour Evaporation (SAFE), Solid Phase Microextraction (SPME), Headspace Sorptive Extraction (HSSE) and Stir Bar Sorptive Extraction (SBSE).

**Figure 2:**
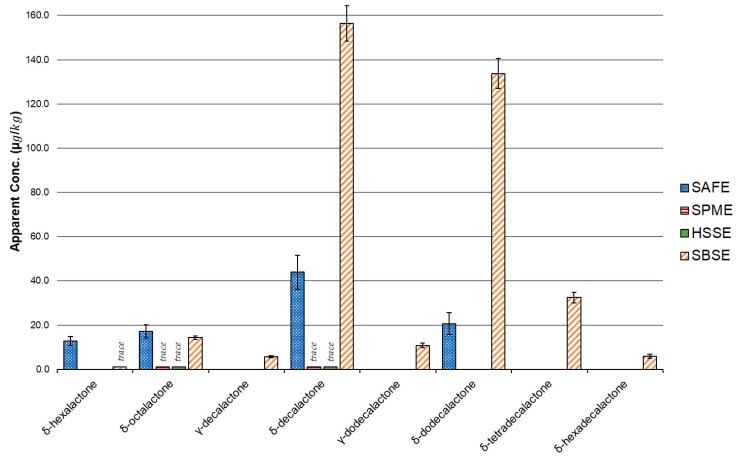
Apparent concentrations of lactones extracted by Solvent Assisted Flavour Evaporation (SAFE), Solid Phase Microextraction (SPME), Headspace Sorptive Extraction (HSSE) and Stir Bar Sorptive Extraction (SBSE). Error bars represent the standard deviations.

Overall, 48 volatiles from nine classes of compounds, alcohols, aldehydes, alkanes, carboxylic acids, ketones, lactones, sulphur compounds, nitrogen compounds, and terpenes were identified in the sheep milk by the four different extraction techniques (Table 1 and Figure 1). The SAFE method detected 20 compounds and the total apparent concentration was 627 µg/kg. The SPME method also detected 20 compounds, while SBSE and HSSE detected 45 compounds and 37 compounds, respectively. The headspace techniques, SPME and HSSE were the least sensitive, with 173 µg/kg and 271 µg/kg of volatiles. The SBSE method was the most sensitive and detected an apparent concentration of 657 µg/kg, mostly due to greater detection of lactones. Among the compounds identified were eight lactones including δ-hexalactone, δ-octalactone, δ-decalactone, γ-decalactone, δ-dodecalactone, γ-dodecalactone, δ-tetradecalactone, and δ-hexadecalactone (Figure 2). Moio et al. had previously reported δ-decalactone and δ-dodecalactone in sheep milk, but to the best knowledge of the authors, this is the first time the other six lactones have been identified in ovine milk. 

**Figure 3:**
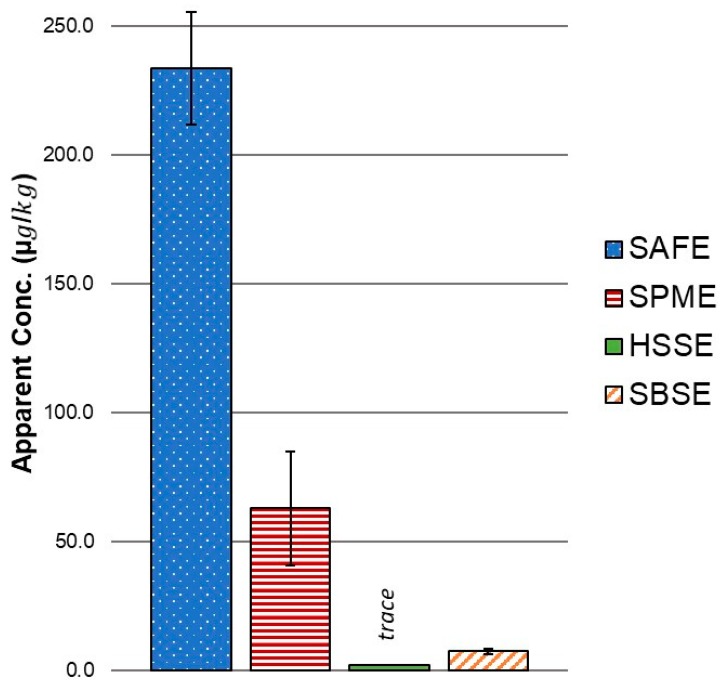
Apparent concentration of dimethyl sulphone extracted by Solvent Assisted Flavour Evaporation (SAFE), Solid Phase Microextraction (SPME), Headspace Sorptive Extraction (HSSE) and Stir Bar Sorptive Extraction (SBSE). Error bars represent the standard deviations.

Compounds with moderate polarity and volatility, such as heptanal, were detected similarly by all four techniques (Table 1). Alkanes were also detected on a similar scale across all four methods. SPME and SAFE were better than the single phased (PDMS) stir bar techniques for extracting small polar analytes such as dimethyl sulphone (Table 1 and Figure 3). SPME was the only technique that detected dimethyl sulphide and pentanal. Carboxylic acids were detected by all four methods but the sensitivity of SPME was noticeably lower than the other three techniques. Ketones were only detected at very low concentrations and only by the PDMS stir bar methods, HSSE and SBSE. Terpenes were detected by all the methods except for SPME. The headspace methods, SPME and HSSE, also detected higher levels of alcohols, especially the relatively polar 1-pentanol, but were only able to detect trace levels of lactones. Conversely, SAFE and SBSE barely detected 1-pentanol but extracted high concentrations of lactones. SAFE was best for the simultaneous extraction of small polar and larger non-polar molecules (e.g., dimethyl sulphone and δ-dodecalactone). SBSE was the most sensitive technique for the lactones. 

### 2.3. Reproducibility

The reproducibility of the methods varied by technique and by compound (Table 1). To compare the reproducibility of the methods, the percent relative standard deviation (RSD’s) for every compound extracted by each method was averaged to provide an average percent relative standard deviation (ARSD) for the method. Based on the average percent relative standard deviations across all compounds SPME and SBSE were the most reproducible with 14.3% and 15.4% ARSD. HSSE and SAFE had higher levels of variability with 26.7% and 49.7% ARSD. In general, the RSD’s were acceptable for most compounds detected by SAFE but the terpenes were less consistent and may have been, at least partially, due to contamination from the environment. For example, limonene and *p*-cymene had RSD’s around 120% and 94% respectively while the RSD for dimethyl sulphone was only 8.3%, heptanal was 13.0%, and the internal standards were all 5.3% or less. HSSE presented similar discrepancies with a minimum RSD of 3.0% for 1-pentanol and a maximum of 99.5% for acetic acid. SPME and SBSE were more consistent overall, with a lower range of RSD values. 

On the low end, SPME delivered RSD’s of 7.0% for decane and 7.6% for pentanal, while the highest RSD was dimethyl sulphone at 35.1%. SBSE was similar to SPME with a minimum RSD of 2.4% for 3,5-octadien-2-one and 42.4% for neophytadiene. Given both the overall average RSD’s and the range of RSD’s, SPME and SBSE were the most reproducible techniques.

## 3. Discussion

### 3.1. Comparison of Method Sensitivity, Selectivity, Reproducibility, and Efficiency

In matrices such as milk where the flavour profile is mild and volatile concentrations are low, recovery of adequate concentrations of volatiles for detection and quantification can be a challenge [11]. The present work optimized and compared four volatile extraction techniques based on their respective benefits and biases. The primary limitations of these techniques were a polarity bias for the PDMS stir bar techniques (HSSE and SBSE) and a volatility bias for the headspace techniques (HSSE and SPME). 

One of the major drawbacks of headspace techniques is that they tend to lack sensitivity for larger, less volatile compounds compared with liquid extraction techniques [30]. The volatility bias was evident in the results of this experiment with HSSE and SPME extracting lower overall concentrations of volatiles from the matrix, largely due to poor of extraction of lactones and carboxylic acids. For example, excluding lactones, SBSE extracted 297 µg/kg and HSSE extracted 271 µg/kg of volatiles in sheep milk. However, with lactones, SBSE extracted more than twice the apparent total concentration of volatiles as the headspace technique, with 657 µg/kg and 271 µg/kg extracted respectively. HSSE and SPME were only able to extract trace levels of δ-octalactone and δ-decalactone. On the other hand, the SBSE technique detected eight lactones and SAFE extracted four lactones. The non-polar bias of the PDMS phase appeared to be beneficial for the extraction of lactones and may explain, at least in part, why SBSE alone was able to extract δ-tetradecalactone and δ-hexadecalactone, due to immersion in the liquid phase for extraction. As expected, due to its lower extraction efficiency, SPME detected lower total apparent concentrations of volatiles from the sheep milk than the other three methods. 

It has been noted in the literature that both highly polar and higher molecular weight volatiles can be well extracted by the SAFE approach depending on the solvent employed, and this study confirmed that finding [31]. SAFE was the only extraction technique that was able to extract high apparent concentrations of both the small polar dimethyl sulphone and the larger less volatile δ-dodecalactone. The non-polar PDMS stir bar methods, HSSE and SBSE, extracted only low concentrations of sulphur compounds while SAFE and SPME excelled. However, the PDMS phase is not exclusively biased against polar compounds. SBSE detected substantial concentrations of dimethyl sulphone while HSSE was only able to detect trace concentrations. However, the concentration detected in both instances was low compared to SAFE and SPME. Possibly due to the broad selectivity of the triple phase DVB/CAR/PDMS fibre, SPME was also the only method to detect pentanal and dimethyl sulphide. There was likely a polarity bias against pentanal and dimethyl sulphide by the PDMS phase of the adsorptive stir bars and the peaks were likely lost under the solvent peak for the SAFE method. 

It can be difficult to discuss reproducibility without also addressing automation and efficiency. Engel et al. found that the SAFE method excelled in terms of efficiency compared to other distillation techniques [31]. Since then, advances in software and hardware have enabled techniques like SBSE, HSSE, and SPME to become simple, highly automated, and efficient methods for extracting volatiles from food matrices. SAFE required significantly more time (approximately 12 h per sample) and effort than the other methods, mostly due to the extensive glassware preparation, manual sample manipulations, and sample concentration steps. SBSE was less efficient than SPME and HSSE for sample preparation due to the manual step of removing non-volatile residues from the PDMS stir bar, but the subsequent GC-MS runs were completed more quickly as a result of the offline sample preparation. HSSE was more efficient than SBSE since the headspace samples did not require the non-volatile residue removal step, but the transfer of the stir bar from the extraction vial to the desorption tube still added time to the sample preparation compared to SPME. SPME was arguably the simplest and therefore the most efficient of the four methods as the volatile extraction and introduction to the GC-MS system could be fully automated by the software and the only manual steps involved were the preparation of the vials and appropriate aliquots of each sample. 

A major benefit of the automation was evident in the reproducibility of the methods. Several factors, such as extensive sample handling, contact with various glassware, and greater overall exposure to the lab environment, may have been reasons that even with careful preparation and procedures, SAFE was the least reproducible of the four extraction methods. SAFE also required a manual concentration step to achieve its sensitivity, which can present further challenges with volatility bias and reproducibility [20]. The reproducibility issues can be corrected for in a targeted analysis where appropriate internal standards may be selected and applied but may present greater challenges for untargeted approaches. The major benefit of SPME is that it is highly automatable and reproducible, making for very efficient analyses when many samples need to be analysed [12]. SBSE was similar in terms of reproducibility compared to SPME for the extraction of volatiles from sheep milk. 

### 3.2. Comparison of Results with Prior Analyses of Sheep Milk Volatiles

Despite the differences in milk supply and processing, the results of this study agreed with previous literature. The four techniques, SAFE, SPME, HSSE, and SBSE, detected between 20 and 48 compounds, well in-line with other studies that identified between 22 and 67 volatiles [8,10]. The range of compound classes was also in agreement with previous studies on fresh milk and included alcohols, aldehydes, alkanes, carboxylic acids, ketones, lactones, sulphur compounds, nitrogen compounds, and terpenes. [4,6,8,9,10,11]. The broader range of compounds identified by Moio et al. can largely be explained by the esters, of which 13 were identified in the raw milk [4]. Esters were not found in the spray-dried New Zealand sheep milk by any of the techniques. Marsili and Miller suggested that the heat load applied to milk by pasteurization may decrease the levels of esters in milk [32]. Studies of pasteurized and unpasteurized milk for cheese-making have also noted a decrease in esters when pasteurized milk was used [33,34]. In addition to other sample differences, the high thermal load involved in pasteurization and subsequent spray-drying the sheep milk may therefore account for some of the differences in the compounds detected between this study and the ones by Moio et al. [4,5,6]. 

Another key difference between this study and previous examinations of sheep milk was the abundance of lactones in the New Zealand sheep milk. In the context of the processing, an increased concentration of lactones is not unexpected. Teng et al. noted that spray-drying could impact the volatile flavour compounds of sheep milk and that it led to increased levels of volatile branched chain fatty acids. It has also been found in past studies that heating fluid milk, in this case by both pasteurization and spray-drying, could lead to the formation of lactones [35]. The propensity of SBSE to extract lactones makes it attractive for the isolation of volatiles from dairy products. δ-decalactone and δ-dodecalactone have been shown to be present in a wide range of dairy products, from milk, non-fat dry milk, and butter to blue cheese [11,21,36]. Lactones have been hypothesized to be important in the volatile profile of sheep milk and their odour-activity and importance has been demonstrated in other dairy matrices [4,6,11,24]. However, the extraction of lactones from milk is inconsistent in the literature, especially when SPME is employed as the isolation technique. While variation in sample origin or processing could explain some of the variability of lactones, it is also possible that lactones are simply not well recovered by SPME or other headspace techniques due to the lower volatility of the lactones. As suggested by Moio et al., it is possible that their importance in sheep milk has been underestimated in the past and this is especially plausible in sheep milk, where the lactones are more likely to partition into the greater lipid phase (5.5–9.27%) compared to cow milk (3.1–5.5%) [4,37]. Buettner et al. also demonstrated the potential of SBSE to extract lactones from human milk and proposed it as a useful tool for the analysis of milk [24].

### 3.3. Overall Comparison

Of the techniques that were applied, SBSE demonstrated the best potential for the evaluation of volatile compounds in sheep milk. In agreement with Buettner et al., it should also be noted that SPME could be a complimentary approach when polar compounds are of interest [24]. SBSE allowed small sample volumes to be prepared, extracted, and analysed efficiently and yielded results with good selectivity, sensitivity, and reproducibility compared to the other approaches. It is widely acknowledged that there is no perfect extraction technique for the analysis of volatiles and each method demonstrated biases according to compound polarity and volatility. As expected, the SPME technique did not extract larger molecular weight compounds well but excelled at extracting small polar compounds like dimethyl sulphone and pentanal. The SBSE technique extracted limited concentrations of small polar compounds, yet excelled at extracting larger molecular weight volatiles, in particular lactones. SAFE was able to extract both small polar compounds and larger more non-polar compounds but the reproducibility and efficiency of the method made it less desirable than the other techniques particularly for applications requiring untargeted analysis of multiple samples. SBSE was the most sensitive method overall and identified δ-hexalactone, δ-octalactone, γ-deca-lactone, γ-dodecalactone, δ-tetradecalactone, and δ-hexadecalactone for the first time in ovine milk. SBSE should be considered an effective tool for the extraction and analysis of volatiles, especially lactones, in sheep milk and dairy products in general.

## 4. Materials and Methods

### 4.1. Chemicals

Sodium chloride and hexane (≥95%) were obtained from Fisher Scientific (Pittsburgh, PA, USA). Sodium sulphate (anhydrous) was obtained from ECP-Analytical (Auckland, New Zealand). 4-Decanone* (≥97.0%) was obtained from Tokyo Chemical Company (Tokyo, Japan). 4-Methyl-2-pentanone* (≥98%) was obtained from BDH Chemicals Ltd. (Poole, UK). Dichloromethane (≥99.9%), methanol (≥99.8%), sodium hydroxide solution (1N), 3-octanone (≥98%), δ-octalactone (98%), γ-decalactone (≥98%), δ-decalactone (≥98%), γ-dodecalactone (≥97%), δ-dodecalactone (≥97%), δ-tetradecalactone (98%) were obtained from Merck (Darmstadt, Germany). A C_7_-C_30_ Saturated Alkane Standard was obtained from Supelco (Bellefonte, PA, USA). Liquid nitrogen, liquid carbon dioxide, hydrogen carrier gas (instrument grade, >99.98%), and nitrogen (instrument grade, >99.99%) were obtained from BOC Ltd. (Auckland, New Zealand).

### 4.2. Spray-Dried Sheep Milk Powder

A single batch of spray-dried sheep milk was obtained from Blue River Dairy (Invercargill, New Zealand). The milk was collected from two separate farms picked up on the same day and pooled in a single transport truck, maintained below 4 °C during transportation, pasteurized, and then spray-dried within 24 h (information provided courtesy of Blue River Dairy). The sheep milk powder had been stored for 16 days sealed in an industry standard plastic lined paper bag containing approximately 9 kg of powder. Upon receipt, the powder was thoroughly mixed and portioned into 50 g aliquots in high-barrier foil vacuum bags. The powders were vacuum sealed on an Audion Audiovac VMS 153 (Derby, UK), labeled, and stored at −18 °C until analysis.

### 4.3. Sample Preparation

A consistent approach was used across all extraction methods for the preparation of the liquid sheep milk from the spray-dried powder. For each analysis, a batch of 250 g of sheep milk was prepared from the sheep milk powder at a concentration of twenty percent solids (w/w). Twenty percent solids was the original average solids content of the source milk on the day of collection at the farm (information provided courtesy of Blue River Dairy). As the milk powder sample was collected during the late-season period, a higher solids content was expected and in agreement with previous studies [37,38]. It was observed that at the high solids content the reconstituted sheep milk tended to foam. To reduce the foaming, the milk was reconstituted in two steps. A 100 g aliquot of deionized water was added to the spray-dried sheep milk powder and mixed thoroughly with a clean spatula to form a slurry, whereupon the remaining 100 g of water could be added with minimal foaming. The sample was covered and mixed for 15 min at room temperature (~20 °C) with a 2.7 cm Teflon coated magnetic stir bar on a Thermolyne Cimarec 2 stir plate (discontinued; Merck, Darmstadt, Germany). A series of three ketones were selected as internal standards for the early, middle, and late eluting compounds based on prior studies in milk [12,16,17,18]. A combined internal standard solution was prepared with 4-methyl-2-pentanone, 3-octanone, and 4-decanone diluted to 500 µg/mL each in methanol. This internal standard solution was used for all extraction techniques. 20 µL was added to 250 g of milk for the HSSE, SBSE, and SPME extractions and 40 µL was added to 250 g of milk for the SAFE technique. The milk was mixed for a further 5 min to incorporate the standards. The required aliquots from the 250 g sample were then taken for each extraction technique.

### 4.4. Equipment and Glassware Preparation

Glass sample vials (20 mL) with septa and screw-top lids were obtained and conditioned prior to use. Glassware was conditioned at 100 °C overnight (approximately 12 h) to remove any residual volatile compounds. Vials, lids, TDU tubes, wires, and septa were conditioned at 130 °C for 2 h prior to analysis. PDMS Twisters^®^ 10 mm × 1 mm film thickness, (Gerstel GmbH, Mulheim an der Ruhr, Germany) and 50/30 µm DVB/CAR/PDMS SPME fibres (Supelco) were conditioned according to manufacturer directions prior to first use and between experiments.

### 4.5. PDMS Stir Bar Extractions (SBSE and HSSE)

For each sample, clean glassware, vials and PDMS stir bars (for both HSSE and SBSE techniques) were prepared. An 8.00 g ± 0.05 g aliquot of the reconstituted sheep milk was transferred by pipette to a 20 mL glass vial. For headspace analyses (HSSE), a clean loop of metal wire was suspended from the septa and the PDMS stir bar was suspended from the loop of wire above the sample of sheep milk. For SBSE, the clean PDMS stir bar was directly added to the sheep milk in the vial by forceps. The vials were then added to a pre-warmed water bath at 35 °C with multi-point magnetic stir plate. The sample was stirred constantly for 90 min. Initial experiments explored extraction times between 60 and 180 min (data not included) and temperatures of 20 °C (room temperature) and 35 °C according to recommended guidelines from the manufacturer for sample size and extraction times [39]. It was found that 90 min at 35 °C gave the best sensitivity while not posing unnecessary risk of artefact formation from excessive thermal load due to high temperatures or very long times. After 90 min, the PDMS stir bars were gently removed with flat, round tipped forceps to avoid damaging the PDMS phase. The PDMS stir bars were rinsed with 3 × 5 mL of deionized water and dried with a lint-free tissue between each rinse, manually removing any sample residues that may have adhered to the stir bar. Extracted and dried PDMS stir bars were placed in clean glass thermal desorption tubes with transport adapters. Samples were desorbed with a programmed temperature ramp from 50 °C to 240 °C at 500 °C/min and held for 5 min. The desorbed sample was cryo-trapped prior to injection in a Gerstel Cooled Injection System (CIS-4) at −60 °C (Gerstel GmbH). The CIS-4 transferred the volatiles to the analytical column by heating from 60 °C to 240 °C at 12 °C/s. The total splitless injection time was 0.8 min. Six sample replications were performed for both the HSSE and SBSE techniques.

### 4.6. Solid Phase Microextraction (SPME)

Clean vials, lids, and septa were obtained for each sample. An 8.00 g ± 0.05 g aliquot of reconstituted sheep milk, spiked with internal standard solution, was transferred by pipette to each sample vial. Samples were placed in a 32-vial tray (VT32-20) on a Gerstel MPS autosampler (Gerstel GmbH) and analyzed by GC-MS. An SPME fibre with a balanced polarity (50/30 μm divinylbenzene/carboxen/polydimethylsiloxane (DVB/CAR/PDMS)) was chosen due to its ability to capture a wide range of volatile compounds from different chemical classes [10,40]. SPME extractions were performed for 60 min at 35 °C and then desorbed in an Agilent split/splitless inlet at 240 °C for 2 min in splitless mode followed by a further 3 min with a purge flow of 50 mL/min. Based on preliminary experiments (data not shown), samples were not held on the autosampler longer than 7 h due to potential for microbial growth. Six sample replications were performed.

### 4.7. Solvent Assisted Flavour Evaporation (SAFE)

SAFE has previously been applied to cow milk in several studies but has not yet been applied to sheep milk. Preliminary experiments (data not shown) investigated diethyl ether and dichloromethane as solvents and assessed a range of solvent to milk ratios based on previous studies [2,11,20,21,22,23]. In the present work, the SAFE apparatus and general operation were as described by Engel et al. [31]. The method selected was adapted from the methods described by Czerny and Schieberle and Moio et al. [6,23]. The SAFE glassware was evacuated and operated at a vacuum less than 6 × 10^−5^ mbar. The sample flask and condenser were equilibrated at 35 °C. A 250 g sample of reconstituted sheep milk was prepared with internal standards and 100 mL of dichloromethane (DCM) was added and mixed for an additional 15 min. The mixture was distilled in the SAFE apparatus over a period of approximately three and a half hours and the distillate was cryogenically trapped with liquid nitrogen in the receiving flask. After distillation, the distillate was salted out and pH adjusted to pH10 with 40.1 g of NaCl and 1N NaOH. After separation, the organic layer was collected and dried with Na_2_SO_4_ (anhydrous) before concentration to 1 mL by kuderna-danish at room temperature under a stream of nitrogen (instrument grade; BOC) at 100 mL/min. A 1 µl aliquot was injected into an Agilent split/splitless inlet in splitless mode at 240 °C. SAFE extractions and instrument injections were each performed in triplicate.

### 4.8. GC-MS Analysis

Gas chromatographic separations were performed with an Agilent Technologies (Agilent Technologies, Beijing, China) 7890B gas chromatograph equipped with a split/splitless inlet as well as a Gerstel CIS4 Cooled Injection System and MPS2 autosampler. On the basis of Imhof and Bosset (1994), many previous studies have employed a non-polar column for the separation of milk volatiles [41]. In preliminary studies (data not shown), it was found that the peak shape for organic acids and ketones was slightly improved on a polar column compared to an HP-5ms and so a SOLGEL-WAX (SGE Analytical Science, Ringwood, Australia) 30 m × 0.25 mm ID × 0.25 µm film thickness analytical column was selected for this study. The analytical column was connected to the mass spectrometer via an inert deactivated fused silica column 1.0 m × 0.1 mm ID (SGE Analytical Science). The separation was conducted with hydrogen carrier gas at 1.6 mL/min constant flow. The analytical column was connected to the mass spectrometer by an Agilent 3-way splitter with makeup gas (part number G3183-60501) operated under constant flow at 1.9 mL/min. The initial oven temperature was 35 °C, held for 4 min, with a 3 °C/min ramp until 100 °C, held for 1 min, followed by a 6 °C/min ramp to 240 °C, and held for 5 min. Compound detection was performed with an Agilent Technologies 5977A Mass in EI mode (70 eV) scanning from 33–300 *m*/*z*. The source and quadrupole were operated at 230 °C and 150 °C, respectively.

### 4.9. Data Analysis

The data was analyzed by MassHunter software (Version B.07.02.1938, Agilent Technologies, Beijing, China). Chromatogram plots were created in R with the “mzR” package [42,43]. Compounds were identified by NIST MS library match (≥80) supported by linear retention indices, as calculated according to Van Den Dool and Kratz from injection of a 1 µg/mL C_7_-C_30_ alkane standard (Supelco) in hexane, and by chemical standards (see Chemicals) [29,44]. The literature retention indices used for comparison were obtained from the NIST Standard Reference Database according to the most similar system and instrument conditions [28]. Semi-quantitation of the analytes in the milk was performed and the results have been presented as “apparent concentrations” as detailed by Gallois and Langlois (1990) [45]. The peak areas of the analytes were normalized to the peak areas of the internal standards and multiplied by the concentration of the internal standard in the reconstituted milk according to Equation (1):

(1) $$Apparent\;Conc.\left(analyte,\;milk\right)\frac{µg}{\mathrm{kg}}=\frac{Peak\;Area\;\left(analyte\right)}{Peak\;Area\;\left(IS\right)}\times Conc\;\left(IS,milk\right)\frac{µg}{\mathrm{kg}}$$

As the study sought only to compare the methods to each other, the actual response factors for each compound were not determined relative to the internal standards as they would not impact the method to method comparisons. Instead a response factor of 1 was assumed for all analytes. Blank injections were performed for each method. Carryover was not observed for blank injections. However, as noted by Buettner et al., the sorptive stir bar methods did appear to pick up small amounts of some compounds from the environment [24]. When a compound did appear in the blank, the peak area in the blank was subtracted from the peak area of the sample prior to normalization.

## Figures and Tables

**Table 1:** Compound identification and apparent concentrations (µg/kg) of volatile analytes in spray-dried New Zealand sheep milk.

		RI ^‡^				SAFE		SPME		HSSE		SBSE		
#	Compound ^†^	Obs	Ref	Method(s) of ID ^¥^	Quant Ion (*m*/*z*)	$\bar{X}$	%RSD	$\bar{X}$	%RSD	$\bar{X}$	%RSD	$\bar{X}$	%RSD	Previous ID Sheep Milk
	***Alcohols***													
1	1-pentanol ^2^	1230	1256	MS, RI	70	*trace*	---	26.7	18.5	11.8	3.0	1.3	6.5	[4,6,10]
2	2-furanmethanol ^2^	1627	1620	MS, RI	98	nd	---	nd	---	6.3	47.8	*trace*	---	[8]
3	phenol ^3^	1961	1965	MS, RI	94	nd	---	nd	---	*trace*	---	*trace*	---	
	*Aldehydes*													
4	pentanal ^1^	935	974	MS, RI	86	nd	---	4.3	7.6	*trace*	---	*trace*	---	[4,6,9,10]
5	hexanal ^1^	1058	1069	MS, RI	82	nd	---	nd	---	*trace*	---	*trace*	---	[4,8,9,10]
6	heptanal ^2^	1163	1165	MS, RI	81	8.6	13.0	5.4	11.1	5.6	15.3	2.5	5.9	[4,6,9,10]
7	octanal ^2^	1266	1267	MS, RI	81	nd	---	nd	---	*trace*	---	0.7	30.1	[4,6,10]
8	nonanal ^2^	1368	1396	MS, RI	98	14.6	54.4	trace	---	7.1	12.9	3.4	12.5	[4,6,10]
9	furfural ^2^	1431	1451	MS, RI	96	nd	---	nd	---	*trace*	---	*trace*	---	[8]
10	2,4-heptadienal ^2^	1460	1469	MS, RI	81	nd	---	nd	---	nd	---	*trace*	---	[10]
11	benzaldehyde ^2^	1485	1485	MS, RI	106	*trace*	---	trace	---	4.7	10.3	1.9	16.0	[4,6]
12	(Z)-2-nonenal ^2^	1502	1534	MS, RI	96	nd	---	nd	---	*trace*	---	*trace*	---	[10]
	***Alkanes***													
13	octane ^1^	800	800	MS, RI	85	nd	---	4.4	8.7	nd	---	nd	---	[9]
14	2,2,4,6,6-pentamethyl heptane ^1^	927	957	MS, RI	99	nd	---	3.8	7.1	7.8	36.9	*trace*	---	
15	decane ^1^	1000	1000	MS, RI	142	nd	---	3.4	7.0	*trace*	---	*trace*	---	[4,6]
16	dodecane ^2^	1200	1200	MS, RI	85	15.5	40.2	9.5	11.1	10.8	26.2	1.1	19.9	[4]
17	tetradecane ^2^	1400	1400	MS, RI	85	nd	---	2.6	13.2	6.7	18.5	1.4	32.4	[4,6]
18	hexadecane ^2^	1600	1600	MS, RI	85	nd	---	trace	---	*trace*	---	0.8	28.8	[4]
19	octadecane ^3^	1800	1800	MS, RI	85	nd	---	nd	---	*trace*	---	9.9	38.2	
	***Acids***													
20	acetic acid ^2^	1425	1440	MS, RI	60	*trace*	---	nd	---	10.3	99.5	4.5	22.1	[8]
21	butanoic acid ^2^	1596	1620	MS, RI	60	nd	---	trace	---	*trace*	---	2.0	32.8	[8,27]
22	hexanoic acid ^3^	1808	1834	MS, RI	60	6.6	127.9	28.7	28.9	19.0	30.4	8.1	11.4	[8,27]
23	octanoic acid ^3^	2017	2050	MS, RI	60	11.1	150.1	18.7	19.7	10.9	13.8	18.8	6.7	[8,10,27]
24	nonanoic acid ^3^	2122	2157	MS, RI	60	19.3	89.8	trace	---	8.9	20.8	5.6	18.7	[27]
25	decanoic acid ^3^	2226	2240	MS, RI	60	16.6	19.3	trace	---	6.9	41.9	90.2	7.5	[8,27]
26	dodecanoic acid ^3^	2435	2449	MS, RI	60	14.1	15.6	nd	---	27.1	26.6	34.5	5.9	[8,27]
27	tetradecanoic acid ^3^	2641	2674	MS, RI	60	nd	---	nd	---	17.2	27.7	28.3	8.4	[8,27]
28	pentadecanoic acid ^3^	2743	2779	MS, RI	60	nd	---	nd	---	5.7	21.3	2.7	26.3	
29	hexadecanoic acid ^3^	2847	2871	MS, RI	60	129.0	139.3	nd	---	91.2	39.9	54.8	17.5	[27]
	***Ketones***													
30	4-methyl-2-pentanone ^1^	981	1008	IS	100	80.0	5.1	40.0	11.8	40.0	30.6	40.0	33.1	
31	3-octanone ^2^	1228	1242	IS	99	80.0	3.5	40.0	12.6	40.0	5.0	40.0	6.1	
32	4-decanone ^3^	1404	---	IS	113	80.0	5.3	40.0	13.0	40.0	8.6	40.0	10.6	
33	2-heptanone ^2^	1160	1160	MS, RI	114	nd	---	nd	---	*trace*	---	0.3	11.6	[4,6,8,10]
34	2-nonanone ^2^	1363	1389	MS, RI	142	nd	---	nd	---	nd	---	0.2	4.9	[4,6,10]
35	3,5-octanedien-2-one ^2^	1487	1521	MS, RI	124	nd	---	nd	---	*trace*	---	0.8	2.4	
36	acetophenone ^2^	1609	1628	MS, RI	120	nd	---	nd	---	1.0	16.9	*trace*	---	
37	2(5H)-furanone ^2^	1710	1712	MS, RI	84	nd	---	nd	---	*trace*	---	*trace*	---	[8]
	***Lactones***													
38	δ-hexalactone ^3^	1747	1751	MS, RI, Std*	70	12.8	16.1	nd	---	nd	---	*trace*	---	
39	δ-octalactone ^3^	1923	1964	MS, RI, Std	99	17.1	17.2	trace	---	*trace*	---	14.3	5.6	
40	γ-decalactone ^3^	2101	2103	MS, RI, Std	85	nd	---	nd	---	nd	---	5.6	8.0	
41	δ-decalactone ^3^	2149	2173	MS, RI, Std	99	43.9	17.7	trace	---	*trace*	---	156.5	5.1	[4,6]
42	γ-dodecalactone ^3^	2331	2353	MS, RI, Std	85	nd	---	nd	---	nd	---	10.8	9.0	
43	δ-dodecalactone ^3^	2380	2395	MS, RI, Std	99	20.7	24.2	nd	---	nd	---	133.8	5.1	[4,6]
44	δ-tetradecalactone ^3^	2609	2701	MS, Std	99	nd	---	nd	---	nd	---	32.5	7.4	
45	δ-hexadecalactone ^3^	2832	---	Std*	99	nd	---	nd	---	nd	---	6.0	14.2	
	***Sulphur compounds***													
46	dimethyl sulphide ^1^	776	777	MS, RI	62	nd	---	2.7	9.7	nd	---	nd	---	[8,10]
47	dimethyl sulphone ^2^	1857	1895	MS, RI	94	233.6	8.3	62.8	35.1	*trace*	---	7.4	14.3	[4,8,10]
	*Terpenes*													
48	D-limonene ^2^	1176	1175	MS, RI	136	4.3	123.5	nd	---	0.8	33.8	0.4	11.4	[10]
49	*p*-cymene ^2^	1248	1253	MS, RI	134	5.4	93.8	nd	---	nd	---	nd	---	[10]
50	neophytadiene ^3^	1914	1915	MS, RI	123	23.4	29.4	nd	---	*trace*	---	15.3	42.4	
	***Other***													
51	*N*,*N*-diethylformamide, ^2^	1391	1413	MS, RI	101	nd	---	d	---	10.6	26.6	.8	18.0	
	***Total Apparent Conc.***					627		173		271		657		

^†^ Compounds: apparent concentration calculated using internal standards, where ^1^ = 4-methyl-2-pentanone, ^2^ = 3-octanone, ^3^ = 4-decanone. ‡ Observed RI calculated according to Van Den Dool and Kratz (1963); Reference RI obtained from the National Institute of Standards and Technology Standard Reference Database Number 69 [28,29]. ^¥^ Method(s) of ID: MS = Mass Spectra library match ≥80, RI = Linear retention index, Std = match to a known chemical standard (see Chemicals), Std* = match determined by retention time match of homologous series of standards (lactones), IS = Internal standard. nd = not detected, --- = could not be determined. trace = detected (Peak area > 0) but signal to noise ratio < 3.0. $\bar{X}$ = average apparent concentration (µg/kg) (SAFE; n = 9, SPME; n = 6, HSSE; n = 6, SBSE; n = 6).
